# Genes Encoding the Glycoprotein Hormone GPA2/GPB5 and the Receptor LGR1 in a Female Prawn

**DOI:** 10.3389/fendo.2022.823818

**Published:** 2022-03-24

**Authors:** Melody Wahl, Tom Levy, Rivka Manor, Eliahu D. Aflalo, Amir Sagi, Joseph Aizen

**Affiliations:** ^1^Department of Life Sciences, Ben-Gurion University of the Negev, Beer-Sheva, Israel; ^2^Department of Life Sciences, Achva Academic College, Arugot, Israel; ^3^National Institute for Biotechnology in the Negev, Ben-Gurion University of the Negev, Beer-Sheva, Israel; ^4^The Faculty of Marine Sciences, Ruppin Academic Center, Michmoret, Israel

**Keywords:** Decapod, Glycoprotein hormone, GPA2/GPB5, Macrobrachium rosenbergii, LGR1, Oocyte development, Reproduction, Vitellogenesis

## Abstract

In vertebrate reproduction, metabolism, growth and development, essential roles are played by glycoprotein hormones, such as follicle-stimulating hormone (FSH), luteinizing hormone (LH) and thyroid-stimulating hormone (TSH), all of which are heterodimers consisting of two subunits, a structurally identical alpha subunit, and a variable beta subunit, which provides specificity. A 'new' glycoprotein hormone heterodimer identified in both vertebrates and invertebrates, including decapod crustaceans, was shown to be composed of the glycoprotein alpha 2 (GPA2) and glycoprotein beta 5 (GPB5) subunits. The putative receptor for GPA2/GPB5 in invertebrates is the leucine-rich repeat-containing G protein-coupled receptor 1 (LGR1). In this study in the giant freshwater prawn, *Macrobrachium rosenbergii*, we identified and characterized the GPA2 *(MrGPA2)*, GPB5 *(MrGPB5)* and LGR1 *(MrLGR1)* encoding genes and revealed their spatial expression patterns in female animals. Loss-of-function RNA interference (RNAi) experiments in *M. rosenbergii* females demonstrated a negative correlation between *MrGPA2/MrGPB5* silencing and *MrLGR1* transcript levels, suggesting a possible ligand–receptor interaction. The relative transcript levels of *M. rosenbergii* vitellogenin *(MrVg)* in the hepatopancreas were significantly reduced following *MrGPA2/MrGPB5* knockdown. *MrLGR1* loss-of-function induced MrVg receptor *(MrVgR)* transcript levels in the ovary and resulted in significantly larger oocytes in the silenced group compared to the control group. Our results provide insight into the possible role of GPA2/GPB5-LGR1 in female reproduction, as shown by its effect on *MrVg* and *MrVgR* expression and on the oocyte development. Here, we suggest that the GPA2/GPB5 heterodimer act as a gonad inhibiting factor in the eyestalk-hepatopancreas-ovary endocrine axis in *M. rosenbergii*.

## Introduction

The vertebrate glycoprotein hormones, such as follicle-stimulating hormone (FSH), luteinizing hormone (LH) and thyroid-stimulating hormone (TSH), produced by the anterior pituitary gland are all heterodimers consisting of two cystine-knot-containing proteins, i.e., a structurally conserved alpha subunit and a variable beta subunit, which provides specificity (1). These vertebrate glycoprotein hormones are essential for reproduction, metabolism, growth and development (1). In addition to these well-studied glycoprotein hormones, sequencing of the human genome revealed a ‘new’ glycoprotein hormone, Thyrostimulin, that is a heterodimer composed of two subunits, designated glycoprotein alpha 2 (GPA2) and glycoprotein beta 5 (GPB5) (2). Similarly to the alpha and beta subunits of the other glycoprotein hormones, GPA2 and GPB5 have conserved cysteine residues, which are important for the formation of key disulfide bonds and hence of the unique cystine knot structure of these hormones (2, 3). Subsequent to the characterization of the vertebrate GPA2 and GPB5 subunits, it was found that these subunits are also are widely distributed in invertebrates, including mollusks (4), annelids (5), urochordates (6), cephalochorates (7), nematodes (8), arthropods (9, 10) and, as recently revealed, also in decapod crustaceans, such as the crayfish, *Procambarus clarkii* (11) and *Cherax quadricarinatus* (12), and the eastern spiny lobster *Sagmariasus verreauxi* (13).

Glycoprotein hormones function by binding to specific leucine-rich repeat (LRR)-containing G protein-coupled receptors (LGRs).These LGRs are characterized by a seven-transmembrane (7TM) helix domain, a large horseshoe-shaped ectodomain – which contains the LRR motif that is responsible for the selective binding of glycoprotein hormones – and a hinge region between the extracellular ectodomain and the anchored 7TM domain, with the hinge region being important for basal receptor conformation and receptor activity (14). Studies on the invertebrates, the fruit fly *Drosophila melanogaster* (15) and the adult mosquito *Aedes aegypti* (16), have shown that the LGR1 receptor is activated by the GPA2/GPB5 heterodimer. Nonetheless, the physiological role of GPA2/GPB5 – in both invertebrates and vertebrates – has not been fully elucidated, although it appears to be pleiotropic (17). Studies of GPA2/GPB5 in invertebrates are limited, and those that have been performed are limited mainly to *D. melanogaster* and *A. aegypti*, in which the heterodimer was shown to be involved in development (18), the hydromineral balance (10), and reproduction (19).

In this study, we focused on the genes encoding GPA2/GPB5 and its putative receptor LGR1 in the decapod crustacean, the giant freshwater prawn *Macrobrachium rosenbergii*, which is one of the best investigated crustacean species by virtue of its high economic value in the aquaculture industry worldwide (20). In decapod crustaceans, the X organ-sinus gland (XO-SG) complex located in eyestalk is a major source of the neuropeptides that regulate multiple physiological processes, including vitellogenesis (21). Vitellogenesis, a crucial process in the ovarian maturation, is characterized by the accumulation of vitellin, which is a yolk protein derived from vitellogenin (Vg). In *M. rosenbergii*, Vg is synthesized in the hepatopancreas (22) and secreted into the hemolymph, from where it is incorporated into the oocytes as a mature vitellin (23). Vg is up taken into the oocytes through endocytosis mediated by the vitellogenin receptor (VgR) (24). It is currently held that ovarian maturation in decapod crustaceans is regulated by two antagonistic neuropeptides—gonad inhibiting hormone (GIH; also known as vitellogenesis-inhibiting hormone, VIH) which is synthesized in the XO-SG complex and inhibits the development of the ovary by inhibiting Vg synthesis, and gonad stimulating factor (GSF), which is thought to be produced by the brain and the thoracic ganglia (TG) (25, 26). Panouse (27) was the first to demonstrate the presence of an ovarian inhibiting factor in the eyestalk of the shrimp *Leander serratus*, since eyestalk ablation during sexual inactivity resulted in the rapid development of the ovaries. It was subsequently shown that implantation of TG or brain tissue or injection of their extracts stimulated vitellogenesis in different crab, crayfish and shrimp species (25, 26, 28–31). Given the evolutionary link between GPA2/GPB5 and the vertebrate gonadotropins FSH and LH, we hypothesized that GPA2/GPB5 is expressed in the central nervous system (CNS; eyestalk and TG) of *M. rosenbergii* and plays a role in reproduction control through the eyestalk-hepatopancreas-ovary endocrine axis. To identify the encoding genes and to study the possible role of GPA2/GPB5 in female reproduction processes, including vitellogenesis, we used transcriptomic databases to characterize the expression patterns of the encoding genes, *MrGPA2*, *MrGPB5* and *MrLGR1* (where *Mr* designates *M. rosenbergii*), combined with *in-vitro* validation in tissues associated with the eyestalk-hepatopancreas-ovary axis. *In-vivo* loss-of-function experiments through RNA interference (RNAi) were performed to elucidate the functionality and the role of the GPA2/GPB5-LGR1 system in *M. rosenbergii* reproduction.

## Materials and Methods

### Animals

*M. rosenbergii* females were collected from the Aquaculture Research Station, Dor, Israel, and were maintained in freshwater tanks at Ben-Gurion University of the Negev (BGU), Beer-Sheva, Israel. The water temperature was kept at 27 ± 2°C, and water quality was assured by circulating the entire volume of water through a bio-filter. Food comprising shrimp pellets (Rangen Inc., Buhl, ID, USA, 30% protein) and frozen marine polychaeta (Ocean Nutrition Ltd., CA, USA) were supplied ad libitum three times a week. The study involves experiments in crustaceans which do not require special permits, nor ethical issues.

### *MrGPA2*, *MrGPB5* and *MrLGR1* Transcripts and Their Deduced Protein Sequences

Genes encoding GPA2 and GPB5 were mined from our existing *M. rosenbergii* transcriptomic libraries (32–34), with the *S. verreauxi* protein sequences, *Sv-GPA2* and *Sv-GPB5* (previously sequenced by Aizen, unpublished data) being used as the queries. A gene encoding *M. rosenbergii* LGR1 was mined using the *A. aegypti* protein sequence *AedaeLGR1* as the query (GenBank accession no. XP_001649032; 10). To deduce the protein sequences, *MrGPA2*, *MrGPB5* and *MrLGR1* were translated by the ExPASy Proteomics Server (http://ca.expasy.org/tools/dna.html), and the longest open reading frame (ORF) was selected for each. Predicted conserved domains were identified using the Simple Modular Architecture Research Tool (SMART) (35). To further characterize MrGPA2, MrGPB5 and MrLGR1, homologous proteins from crustaceans, insects and human were selected for sequence alignment (**Table 1**). The ClustalW multiple alignment analyses were conducted with the Molecular Evolutionary Genetics Analysis MEGAX (36).

**Table 1:** Proteins used for sequence alignments.

Species	Protein	Accession number
*Aedes aegypti*	GPA2	BN001241
	GPB5	BN001259
	LGR1	KF711859
*Drosophila melanogaster*	GPA2	NP_001104054.2
	GPB5	NP_001104335.1
	LGR1	AAB07030
*Homo sapiens*	GPA2	NP_570125.1
	GPB5	NP_660154.3
	thyrotropin receptor	NP_000360
	follicle-stimulating hormone receptor	NP_000136
	lutropin-choriogonadotropic hormone receptor	NP_000224
*Sagmariasus verreauxi*	GPA2	NA
	GPB5	NA

### Temporal Expression Patterns in Early Developmental Stages

Our existing *M. rosenbergii* embryo library (34) provides *in silico* temporal expression patterns for *MrGPA2*, *MrGPB5* and *MrLGR1* at different embryonic stages (day 1, day 3, day 5, day 11 and day 17) in all-male or all-female embryonic populations. These populations were produced in our laboratory using previous biotechnologies for all female population (37, 38) or for all male population (39, 40). To expand the temporal expression pattern to include the later developmental stages zoea 4 (larva) and post-larva 1 (PL; one day after metamorphosis), RNA was extracted from female larvae, male larvae, female PLs and male PLs (4 replicates per stage) using the EZ-RNA Total RNA Isolation Kit (Biological Industries) according to the manufacturer’s instructions. cDNA was prepared by a reverse-transcriptase reaction using the qScript cDNA kit (Quanta BioSciences) containing 1 μg extracted total RNA. qPCR was conducted to obtain the relative quantification of *MrGPA2*, *MrGPB5* and *MrLGR1* transcript levels using specific primers (**Table 2**) and Universal ProbeLibrary Probes (Roche; **Table 2**) with the SensiFAST Probe Hi-ROX Mix (BIOLINE). *Mr18S* (GenBank accession no.GQ131934) was used as a normalizing gene (**Table 2**). The qPCR reactions were performed in the QuantStudio Real-Time PCR System, Applied Biosystems (Foster City, CA, United States).

**Table 2:** Primers and probes from Roche probe library used for qPCR.

	F primer	R primer	Probe
***MrGPA2***	GACCACGGGAGCTGATCTT	CTCTTCTTAATACTTTTTGCAGTGGA	17
***MrGPB5***	CTGGGAACTTCAAAGGAACG	AAATCTTCTGTCACAGCCCTTT	4
***MrLGR1***	CACTCCGATCTCACCGTAGC	CAGCAGGCAAAGTCTGTGAA	91
***MrVg***	TTTGAAGTTAGCGGAGATCTGA	TTCGAATTTGCGCAGTCTTT	144
***MrVgR***	GATAAGCAACCCGCAGGAG	CTGAGGAACCTCGACTACGG	91
***Mr18S***	CCCTAAACGATGCTGACTAGC	TACCCCCGGAACTCAAAGA	152

### Spatial Expression Pattern in the Tissues of Adult Females

Eyestalk, ovary, hepatopancreas, TG, and muscle tissue were dissected from *M. rosenbergii* females (n = 12), and RNA was extracted from each tissue as described above. cDNA was synthesized and relative quantification of *MrGPA2*, *MrGPB5* and *MrLGR1* transcript levels was performed using qPCR with the relevant specific primers and probes (**Table 2**), as described above.

### dsRNA Preparation

Two PCR products were generated for each gene (*MrGPA2*, *MrGPB5* and *MrLGR1*) using a T7 promoter anchor (T7P; 5’-TAATACGACTCACTATAG GG-3’) attached to one of the two primers used to amplify each product (**Table 3**). The primed products were used as templates for RNA synthesis. dsRNA was prepared using Thermo Scientific TranscriptAid T7 High Yield Transcription Kit, according to the manufacturer’s instructions. The sense and antisense strands were hybridized by heating to 70°C for 15 min and to 65°C for 15 min, followed by incubation at room temperature for 30 min. RNA was quantified and diluted to 1 μg/μL, and quality was assessed on 1.5% agarose gel. dsGFP was used as control exogenous dsRNA and was synthesized as previously described by Ventura et al. (41). The dsRNA was maintained at -80°C until used.

**Table 3:** Gene-specific primers with T7 promoter site at the 5’ of one primer used as templates for dsRNA synthesis.

	F primer	R primer
***MrGPA2* sense**	T7P -TCGACGTATTCGTTTCCTCA	GATGACCTGGTGAGGGTTGT
***MrGPA2* antisense**	TCGACGTATTCGTTTCCTCA	T7P-GATGACCTGGTGAGGGTTGT
***MrGPB5* sense**	T7P-TCTCTCCACCCTCGAATGTC	ACCTCTGGGCATTTTGGCGCGAG
***MrGPB5* antisense**	TCTCTCCACCCTCGAATGTC	T7P-ACCTCTGGGCATTTTGGCGCGAG
***MrLGR1* sense**	T7P-TGTACGCCATTCTCACGAAG	TTGTCTGACAGCGTGAGTCC
***MrLGR1* antisense**	TGTACGCCATTCTCACGAAG	T7P-TTGTCTGACAGCGTGAGTCC

### Short-Term Loss-of-Function Efficiency of RNAi

Before the actual performance of functional experiments with a candidate gene, we performed a short-term experiment to evaluate ds*MrGPA2*, ds*MrGPB5* and ds*MrLGR1* silencing efficiency. For such a short-term loss of function experiment, previtellogenic females (11 ± 0.4 g) were divided into three groups. Ten females were injected with a mix of ds*MrGPA2* and ds*MrGPB5*, 9 females were injected with ds*MrLGR1*, and 9 females served as controls and were injected with an exogenous dsRNA (ds*GFP*). Since this research does not follow the hormone at its hormonal level such as its heterodimerization, knockdown of *MrGPA2* and *MrGPB5* together was used to increase the certainty of affecting at the protein level. All animals were injected twice (on days 1 and 3) in the abdominal muscle with 5 µg of dsRNA per gram of body weight. Two days after the second injection, the animals were dissected, and total RNA was extracted from the eyestalk, TG, ovary and hepatopancreas of each animal, as described above. To validate the silencing efficiency, cDNA was synthesized, and the relative transcript levels of *MrGPA2*, *MrGPB5* and *MrLGR1* were quantified using qPCR, as described above. The relevant target tissues for the RNAi further analyses were chosen according to the spatial expression results in adult females.

### Long-Term Loss-of-Function Experiment

Having established the efficiency of RNAi-based silencing in the above-described short-term experiment, we set out to perform a long-term RNAi loss-of-function experiment to investigate the role of the GPA2/GPB5-LGR1 system in female reproduction, and more specifically its relation to the eyestalk-hepatopancreas-ovary axis. To this end, 36 previtellogenic females (12.6 ± 0.2 g) were divided into three equal groups: two treatment groups (n = 12, injected with a mix of ds*MrGPA2 +* ds*MrGPB5*, and n = 12, injected with ds*MrLGR1*) and a control group (n = 12, injected with dsGFP). Each animal was injected (as described above) once a week over an eight-week period. One week after the last injection, 6 females in each treatment group and 4 females in the control group were still alive. Each female was weighed and dissected. The whole gonad was dissected out and weighed for calculation of the gonadosomatic index (GSI) (gonad weight as a proportion of total body weight; $\%GSI=\frac{Gonad\;weight\;(g)}{Total\;body\;weight\;(g)}\times 100$ ). RNA was extracted from the eyestalk, ovary, hepatopancreas and TG tissue of each animal, and cDNA was synthesized for qPCR. Relatively small prawns were used to ensure the uniformity of such experiments with respect to reproductive state of the ovary. To enable investigation of a possible relationship between the GPA2/GPB5-LGR1 system and vitellogenesis, the relative transcript levels of *MrVg* in the hepatopancreas and *MrVgR* in the ovary were quantified, as described above.

### Histology and Oocyte Diameter Measurements

Gonads were fixed for histology in 4% buffered formalin for 48 h, followed by dehydration using increasing ethanol concentrations (70, 80, 90, and 100%). Samples were then incubated in xylene and embedded in Paraplast (Kendall). Histological sections of the ovaries were stained with hematoxylin and eosin (H&E) for morphological observations, as previously described (42). The diameters of representative oocytes (n = 5) for each gonad were measured using ImageJ software (43). To verify consistency of the measured area between different slides, the diameters were measured only in oocytes in which the nucleus was visible. The average oocyte sizes were compared between the control and treatment groups in the long-term loss-of-function experiment.

### Statistical Analyses

All data was logarithmically transformed to facilitate proper statistical analysis. For the spatial expression patterns of *MrGPA2*, *MrGPB5* and *MrLGR1* in females, the comparisons of the relative transcript levels between tissues were analyzed using one-way ANOVA, followed by *post hoc* Tukey’s HSD test. According to the one-way ANOVA assumptions, the residuals’ normality was tested using the Shapiro-Wilk test, and the homogeneity of variances was tested using Levene’s test. For the relative quantification by real time PCR in the short- and long-term loss-of-function experiments, as well as for the *MrVg* and *MrVgR* relative transcript levels, GSI and oocyte diameters, data was compared and analyzed using a *t*-test. All statistical analyses were performed using Statistica v13.5 software (StatSof Ltd., Tulsa, OK, USA).

## Results

### Identification of *MrGPA2*, *MrGPB5* and *MrLGR1*

**Figure 1:**
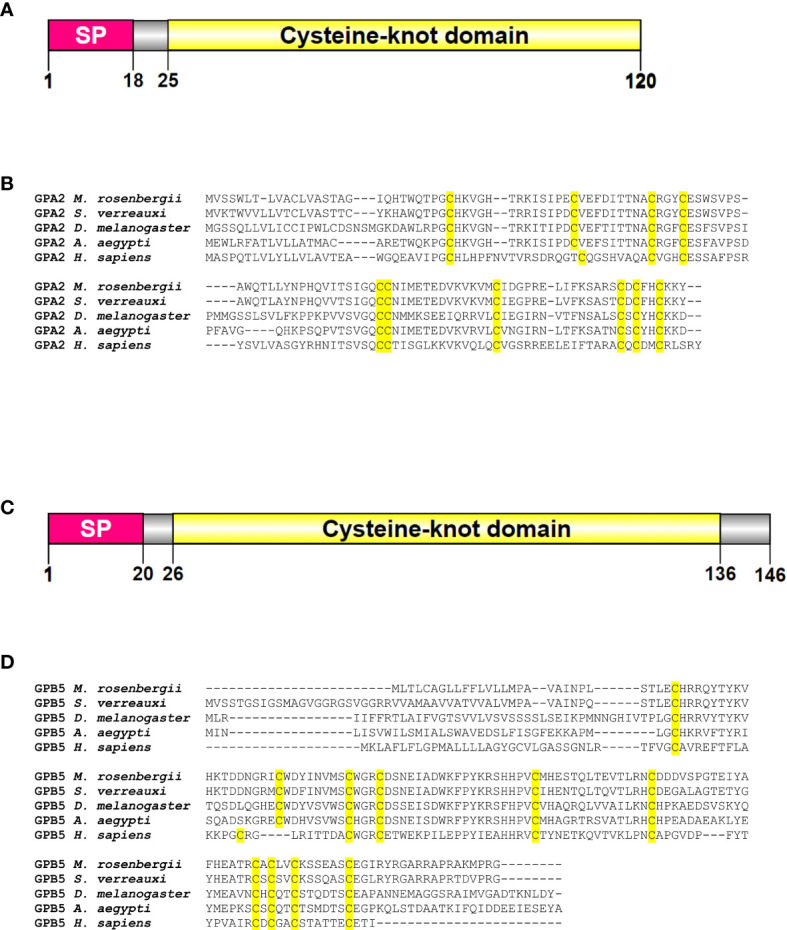
*M. rosenbergii* glycoprotein hormone subunits and deduced protein sequences. Linear models of the **(A)** GPA2 and **(C)** GPB5 proteins of *M. rosenbergii* showing the conserved predicted domains of the proteins containing a signal peptide and a cysteine-knot domain. The location of the amino acids in the protein is scaled. Multiple sequence alignment of **(B)** GPA2 and **(D)** GPB5 from *M. rosenbergii*, *S. verreauxi*, *A. aegypti*, *D. melanogaster* and *H. sapiens* demonstrates the conservation of key cysteine residues (highlighted in yellow).

Searches of the transcriptomic libraries revealed *MrGPA2* (2,328 bp) and *MrGPB5* (1,941 bp) transcripts with predicted ORF encoding translation products of 120 and 146 amino acids, respectively (**Supplementary Material**). The deduced protein structures of MrGPA2 and MrGPB5, according to their ORFs, contain a signal peptide and a cystine-knot domain (**Figures 1A, C**). Sequence alignments of *M. rosenbergii*, *S. verreauxi*, *D. melanogaster, A. aegypti* and *Homo sapiens* confirmed the conservation of the key cysteine residues of GPA2 and GPB5 that are essential for the formation of the disulfide bridges involved in the characteristic cystine-knot structure (3) (**Figures 1B, D**).

**Figure 2:**
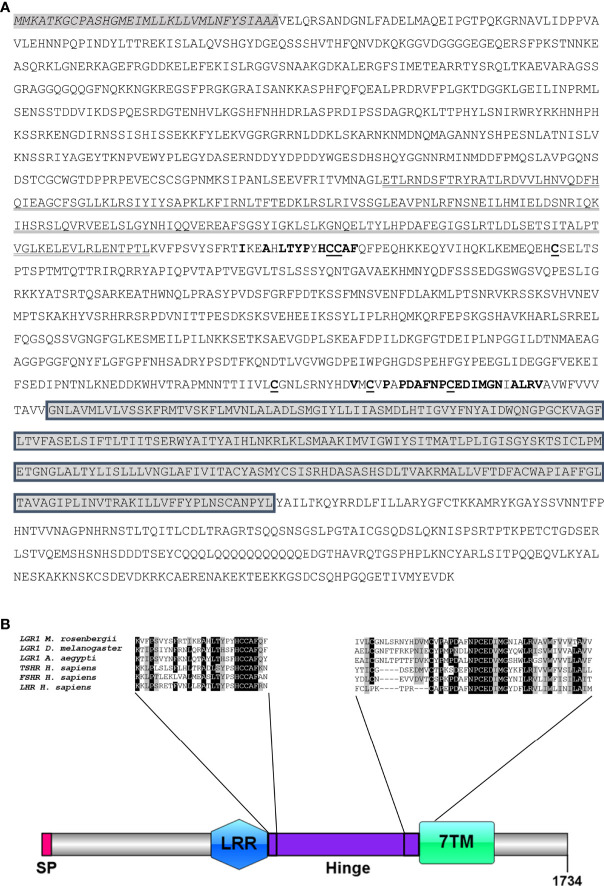
MrLGR1 open reading frame. **(A)** Deduced amino acid sequence of MrLGR1 and predicted domain sites. Beginning at the N terminus; signal peptide (italicized, gray background), leucine rich repeat domain (bold), hinge region consensus sequences (red and bold), six cysteines in the hinge region (underlined), and the trans-membrane domain (gray boxes). **(B)** Scaled illustration of MrLGR1 conserved domains, including: signal peptide (SP), leucine-rich repeats (LRR), hinge region (Hinge) and the 7 transmembrane helices (7TM). Multiple sequence alignment of the hinge region of MrLGR1, insect LGR1 and the *H. sapiens* glycoprotein receptors presents the consensus sequences at the beginning and the end of the hinge.

Searches of the transcriptomic libraries also revealed an *MrLGR1* (5,391 bp) transcript with a predicted ORF of 1,734 amino acid (**Figure 2A**; **Supplementary Material**). LGR1 is a type A LGR in that it contains LRRs (typically 7–9) and a long hinge region in its ectodomain in addition to a G protein-coupled receptor (GPCR)-conserved 7TM domain (**Figure 2A**). The hinge region occupies the sequence between the LRRs and the transmembrane domain and is specific for each of the three main types of LGRs (14). The type A hinge region contains the consensus sequence L-XX-A-X-LTYP-X-HCCAF at the beginning of the hinge and the consensus sequence V-X-C-X-P-X-PDAFNPCEDIMGY-X-FLRV at the end of the hinge (**Figure 2B**). Alignment of the MrLGR1 amino acid sequence with LGR1 from insects and *H. sapiens* glycoprotein hormone receptors (TSHR, FSHR and LHR) showed similar domain compositions, with only slight differences (**Figure 2B**). In addition, the hinge region of MrLGR1 contains six cysteine residues (Cys_770_, Cys_771_, Cys_798_, Cys_1201_, Cys_1213_ and Cys_1223_; **Figure 2A**), namely, two cysteines in each of the two consensus sequences and two more cysteines in the hinge region, with these six cysteines probably forming three disulfide bridges stabilizing the entire structure.

### Temporal Expression Patterns

**Figure 3:**
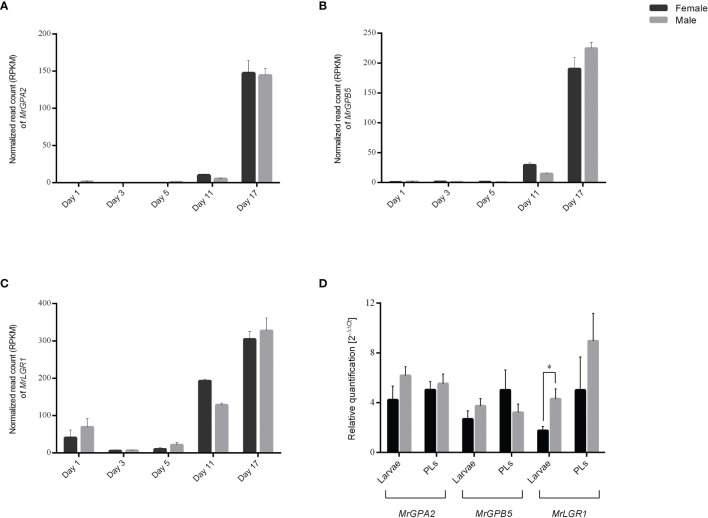
*In silico* and *in vitro* temporal expression patterns. **(A–C)** *In-silico* temporal expression patterns in *M. rosenbergii* embryonic stages for **(A)** *MrGPA2*, **(B)** *MrGPB5* and **(C)** *MrLGR1*. The number of mapped reads per sample (i.e., day 1, day 3, day 5, day 11 and day 17 in males and females) was normalized by reads per kilobase of transcript per million mapped reads (RPKM), dividing it by the total number of reads from that sample and multiplied by 1 × 10^6^. **(D)** *In-vitro* temporal expression patterns. Relative quantification of *MrGPA2*, *MrGPB5*, and *MrLGR1* in male and female larvae and post-larvae (PLs). Asterisks represent the statistically significant differences (*P* < 0.05, *t* test).

To study the *in silico* expression patterns of *GPA2*, *GPB5* and *LGR1* in early *M. rosenbergii* development during the embryo stages, an embryo transcriptomic library at five different stages in males and females (34) was used. The transcripts, which were found to be non-sexually biased, indicated high expression levels of *MrGPA2* and *MrGPB5* on day 17 (**Figures 3A, B**) and enhanced expression of *MrLGR1* on days 11 and 17 (**Figure 3C**). In developmental stages beyond embryos (larva and PL), no sexually biased differences were found in *MrGPA2* and *MrGPB5* relative transcript levels. In contrast, *MrLGR1* relative transcript levels were found to be significantly higher in male than in female larvae (*t*_6_ = -3.01, *P* < 0.05), although the relative transcript levels in PLs did not significantly differ between the sexes (**Figure 3D**).

### Spatial Expression Patterns in Female Prawns

**Figure 4:**
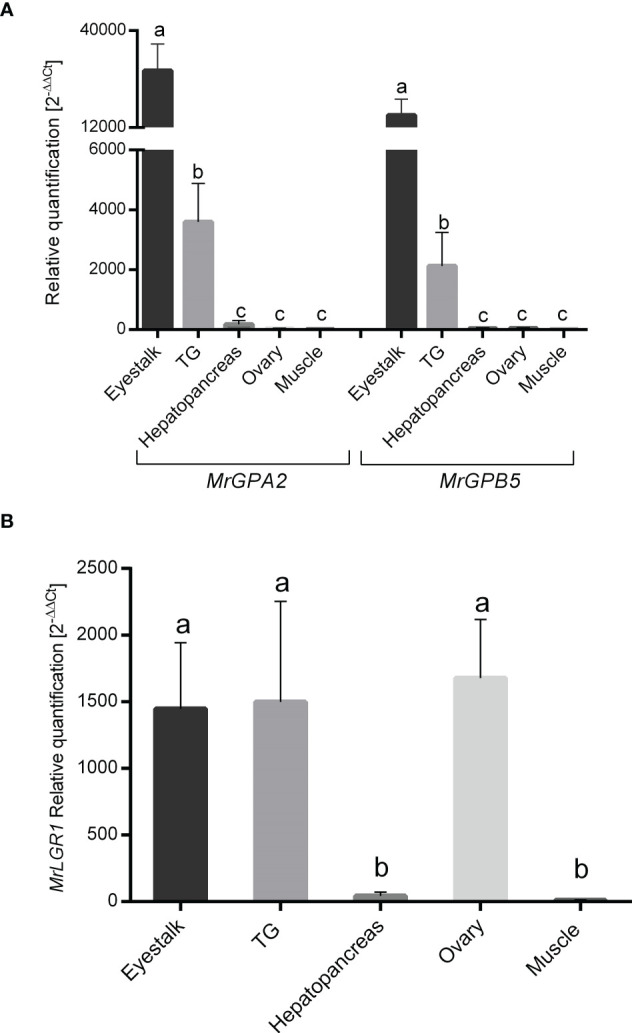
Spatial expression patterns in *M. rosenbergii* females. Relative quantification in the eyestalk, thoracic ganglion (TG), hepatopancreas, ovary and muscle of **(A)** *MrGPA2* and *MrGPB5* and **(B)** *MrLGR1* in adult females (n = 12). Error bars represent standard error of the means and different letters on the bars indicate statistically significant differences (*P < 0.05*, one-way ANOVA *post hoc* Tukey’s test).

Relative transcript levels of *MrGPA2* and *MrGPB5* exhibited similar spatial expression patterns for the two genes, but with significantly different values between the different tissues for each gene (*P* < 0.05, one-way ANOVA with *post hoc* Tukey’s test). Specifically, significantly higher expression levels were found in the eyestalk and the TG than in the other tissues (hepatopancreas, ovary and muscle), with the levels in the eyestalk being approximately sevenfold higher than those in the TG (**Figure 4A**). *MrLGR1* relative transcript levels were found to be significantly higher in the eyestalk, TG and ovary compared to the hepatopancreas and muscle (*P* < 0.05, one-way ANOVA with *post hoc* Tukey’s test) (**Figure 4B**).

### MrGPA2, MrGPB5 and MrLGR1 Knockdown Effects

**Figure 5:**
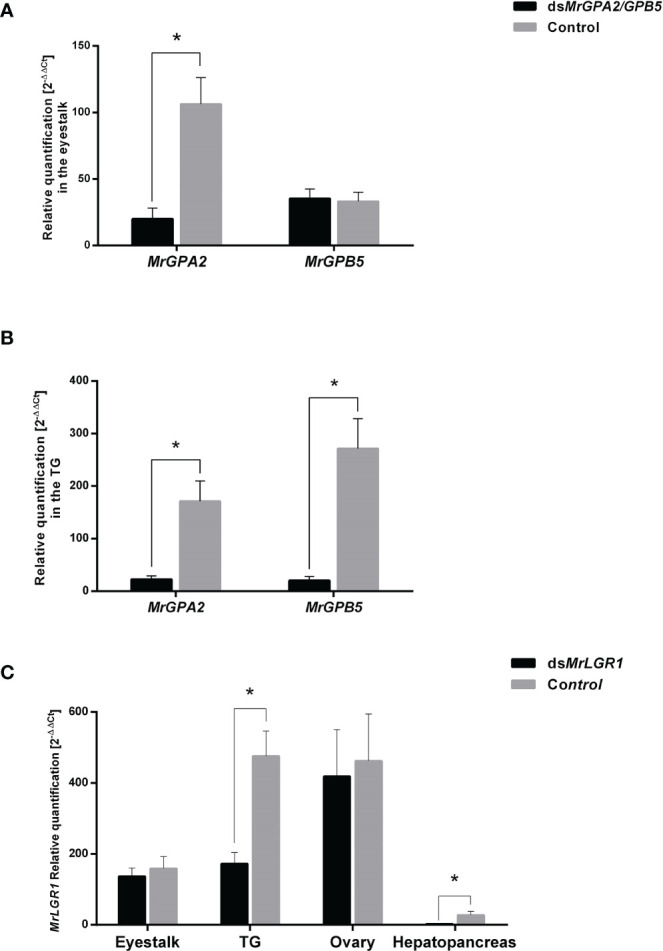
Short-term loss of function through RNAi. Relative quantification of *MrGPA2* and *MrGPB5* in **(A)** the eyestalk and **(B)** the thoracic ganglia (TG) of *M. rosenbergii* females injected with mix of ds*MrGPA2* and ds*MrGPB5* (n = 10) or with dsGFP (control; n = 9). **(C)** Relative quantification of *MrLGR1* in the eyestalk, TG, ovary and hepatopancreas of females injected with ds*MrLGR1* (n = 9) or with dsGFP. Error bars represent standard error of the means. Asterisks indicate statistically significant differences (*P* < 0.05, *t* test).

To test the silencing efficiency of RNAi using ds*MrGPA2*, ds*MrGPB5* and ds*MrLGR1*, a short-term experiment was performed. In the eyestalk, *MrGPA2* relative transcript levels decreased significantly by 81.1% (*t*_16_ = 4.32, *P* < 0.01) in the ds*MrGPA2-*injected group compared to the control. In contrast, for *MrGPB5*, the difference in relative transcript levels between the ds*MrGPB5-*injected and control groups was negligible (*t*_17_ = -0.17, *P* > 0.05) (**Figure 5A**). Specific knockdown of *MrGPA2* and *MrGPB5* resulted in a significant decrease in their expression in the TG, with an efficiency of 92.4% (*t*_15_ = -5.24, *P* < 0.01) and 86.6% (*t*_15_ = -8.31, *P* < 0.01), respectively (**Figure 5B**). Relative transcript levels of *MrLGR1* in the TG and hepatopancreas were significantly reduced by 63.8% (*t*_14_ = 3.6, *P* < 0.05) and 89.6% (*t*_14_ = 3.9, *P* < 0.05) in the ds*MrLGR1-*injected group compared to the control, but levels in the eyestalk and the ovary did not differ significantly between the silenced and control groups (*P* > 0.05; *t*_16_ = 0.28 and *t*_11_ = 0.07, respectively) (**Figure 5C**).

**Figure 6:**
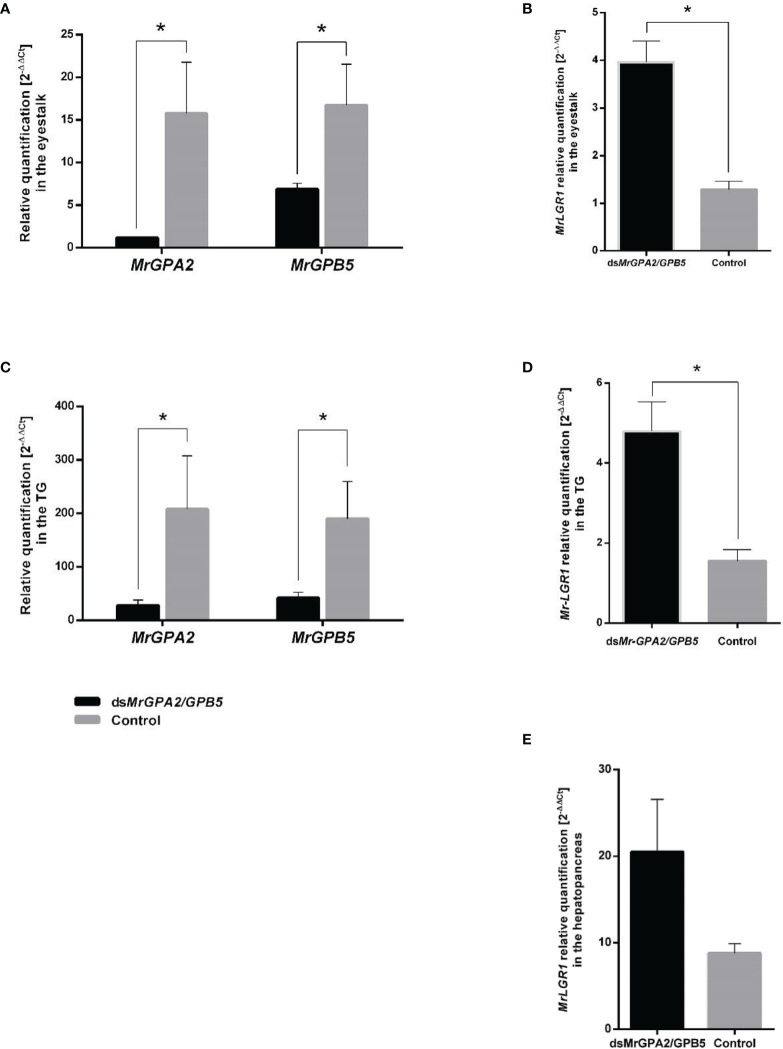
Long-term *MrGPA2* and *MrGPB5* loss of function through RNAi. Females were injected with a mixture of ds*MrGPA2* and ds*MrGPB5* (n = 6) or with dsGFP (control; n = 4). Relative transcript levels of *MrGPA2* and *MrGPB5* in **(A)** the eyestalk and **(C)** the thoracic ganglia (TG). Mr*LGR1* relative quantification following *MrGPA2/GPB5* knockdown in **(B)** the eyestalk, **(D)** TG and **(E)** the hepatopancreas. Error bars represent standard error of the means. Asterisks indicate statistically significant differences (*P* < 0.05, *t* test).

**Figure 7:**
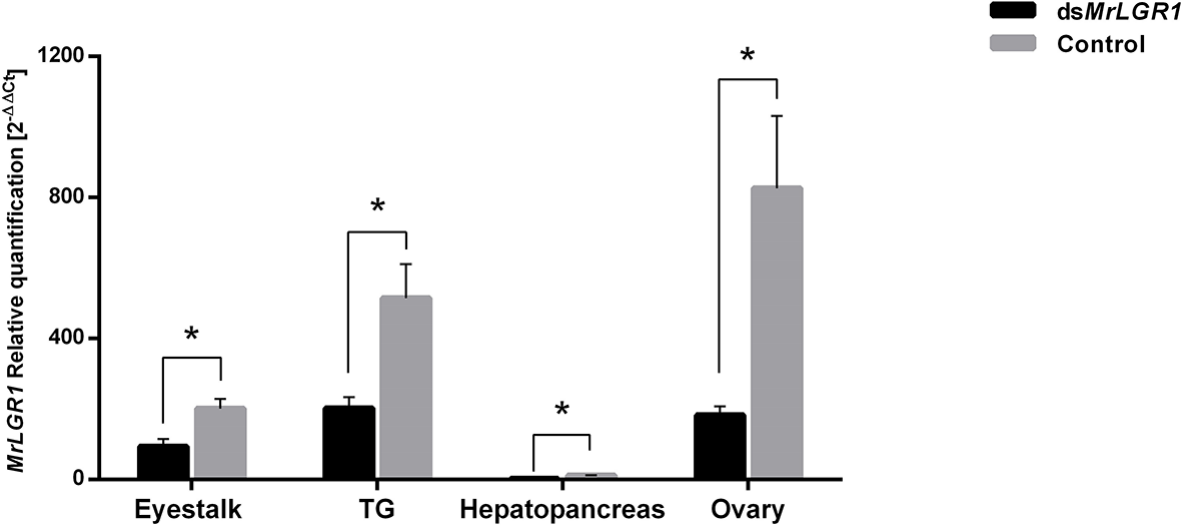
Long-term *MrLGR1* loss of function through RNAi. Females were injected with ds*MrLGR1* (n = 6) or with dsGFP (control; n = 4). *MrLGR1* relative transcript levels in the eyestalk, TG, hepatopancreas and ovary. Error bars represent standard error of the means. Asterisks indicate statistically significant differences (*P *< 0.05, *t* test).

During the eight-week long-term experiment, relative transcript levels of *MrGPA2* and *MrGPB5* in the eyestalk were significantly reduced. This was evident by measurements at the end of the above period showing 88.01% (t_7_ = 23.68, *P* < 0.01) and 59.9% (t_8_ = 3.44, *P* < 0.01) reduction in the *MrGPA2* and *MrGPB5*, respectively, in the ds*MrGPA2/MrGPB5*-injected group vs. the control group (**Figure 6A**). Similar findings were obtained for the TG, i.e., a significant reduction for the silenced vs. the control groups of 86.6% (*t*_8_ = -3.14, *P* < 0.05) and 79.1% (*t*_8_ = -3.46, *P* < 0.01), respectively (**Figure 6C**). For *MrLGR1*, the relative transcript levels were significantly higher – by approximately threefold – in the *MrGPA2/MrGPB5*-silenced group compared to the control group in both the eyestalk (*t*_8_ = -4.71, *P* < 0.01) (**Figure 6B**) and the TG (*t*_7_ = -2.97, *P* < 0.05) (**Figure 6D**). However, when quantified, the transcript levels of *MrLGR1* in the hepatopancreas was not significantly different in the *MrGPA2/MrGPB5*-silenced group and the control (**Figure 6E**). *MrLGR1* knockdown resulted in significantly reduced relative transcript levels of *MrLGR1* in the ds*MrLGR1*-injected group in the eyestalk, TG, hepatopancreas and ovary—by 53.5% (*t*_8_ = 3.27, *P* < 0.05), 81.8% (*t*_8_ = 3.35, *P* < 0.05), 82.7% (*t*_8_ = 1.03, *P* < 0.05) and 77.9% (*t*_8_ = 5.62, *P* < 0.01), respectively (**Figure 7**).

**Figure 8:**
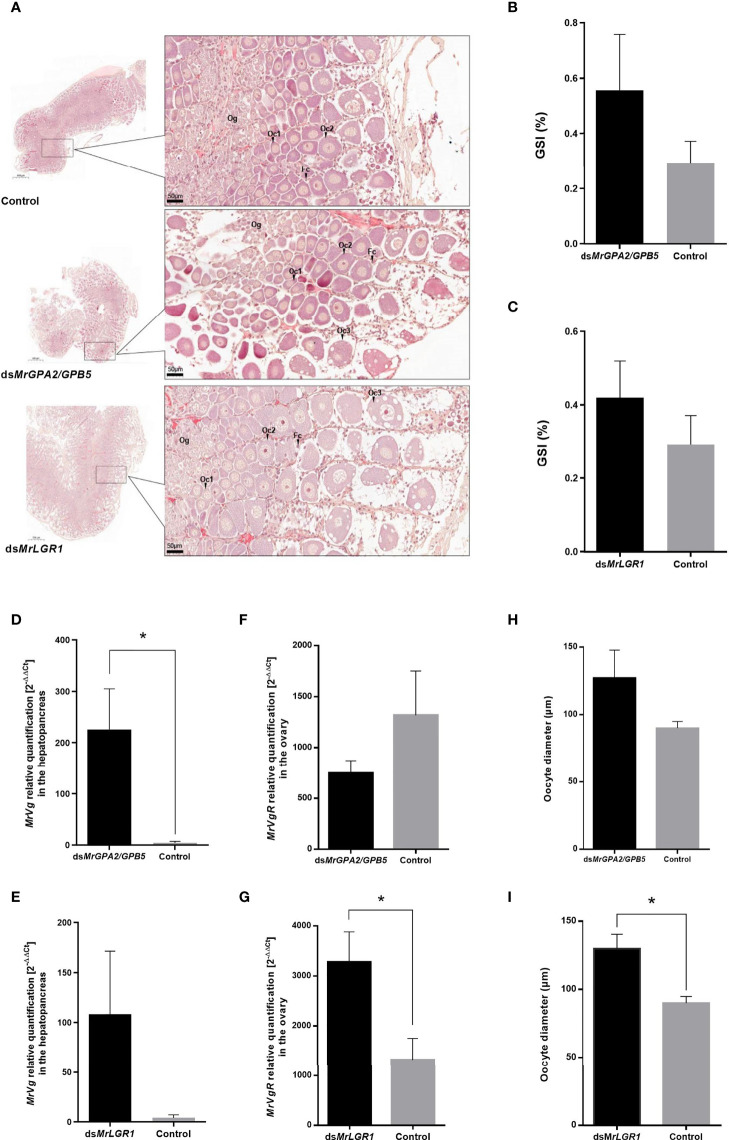
Reproductive effects following long-term *MrGPA2/GPB5* and *MrLGR1* loss of function experiments. **(A)** Representative histological sections of ovaries of control (dsGFP injected; top), ds*MrGPA2/GPB5*-injected and ds*MrLGR1-*injected (bottom) females. *MrGPA2/GPB5* (top panels) and *MrLGR1* (bottom panels) knockdown effects on **(B, C)** *M. rosenbergii* gonado-somatic index (GSI), **(D, E)** vitellogenin (*MrVg*) relative transcript levels in the hepatopancreas, **(F, G)** vitellogenin receptor (*MrVgR*) relative transcript levels in the ovary and **(H, I)** oocyte diameter. Error bars represent standard error of the means. *Statistically significant difference (*P* < 0.05, *t* test).

Histological sections of the ovaries (**Figure 8A**) enable morphological examination of the oocyte stages (classified according to 44). The ovaries of all studied females (control, ds *MrGPA2/MrGPB5*-injected and ds*MrLGR1*-injected) contain predominantly oogonia (Og), early previtellogenic oocytes (Oc_1_) and late previtellogenic oocytes (Oc_2_). However, early-vitellogenic oocytes (Oc_3_) seem to be more abounded in ovaries sections of *MrLGR1-*silenced females than those of the control group (**Figure 8A**). GSI (%) values of less than 1 for all females indicate on similar previtellogenic stage of the treatments and control groups. These values did not significantly differ between the control (0.29 ± 0.08) to the *MrLGR1-*silenced (0.42 ± 0.10) group nor the *MrGPA2/MrGPB5-*silenced (0.56 ± 0.20) group (**Figures 8B, C**) (*P* > 0.05; *t*_8_ = -0.91 and *t*_8_ = -1.6, respectively).

To study the reproductive effects of knockdown of *MrGPA2/MrGPB5* and of *MrLGR1* in the context of the eyestalk-hepatopancreas-ovary axis, the relative transcript levels of Vg – as a good indicator of endocrine control of female reproduction (45) – in the hepatopancreas were quantified. It is noteworthy that even in the previtellogenic females, ds*MrGPA2/MrGPB5* treatment had a significant effect (*t*_5_ = -5.46, *P* < 0.01) which increases Vg relative transcript levels. On the other hand, ds*MrLGR1* treatment was not statistically significant (*t*_6_ = -2.12, *P* > 0.05) (**Figures 8D, E**). Contrary, *MrLGR1* knockdown caused significant induction of *MrVgR* expression levels in the ovaries of the silenced group compared to the control (*t*_8_ = -2.96, *P* < 0.01), while following *MrGPA2/MrGPB5* knockdown, no significant difference was obtained between the silenced and control groups (*t*_8_ = -1.48, *P* > 0.05) (**Figures 8F, G**). Average oocyte diameters in females of the *MrGPA2/MrGPB5*-silenced [127.09 ± 46.16 (SD) µm] and control [89.85 ± 8.45 (SD) µm] groups did not differ significantly (*t*_8_ = -1.59, *P* > 0.05) (**Figure 8H**). In contrast, *MrLGR1* knockdown resulted in significantly larger oocytes [129 ± 23.3 (SD) µm; *t*_8_ = -2.88, *P* < 0.05] in the ds*MrLGR1*-injected group vs. the control group (**Figure 8I**).

## Discussion

In both vertebrates and invertebrates, reproductive success relies on coordinated control of the reproductive cycle through endocrine axes. Extensive studies have shown that the classical glycoprotein hormones, including FSH, LH and TSH, are structurally and functionally conserved in vertebrates (e.g., 1, 9, 46–49), but the physiological role of GPA2/GPB5 remains elusive—in both vertebrates and invertebrates (17). In this study, we report the identification of genes encoding GPA2, GPB5 and LGR1 in *M. rosenbergii*, their temporal expression patterns in early developmental stages (embryo, larvae and PLs), and their spatial expression patterns in different tissues of adult females. Both MrGPA2- and MrGPB5-deduced proteins exhibit the 10 conserved cysteine residues that are typically found in vertebrate and invertebrate GPA2/GPB5 amino acid sequences (9) and that are essential for disulfide binding and loop formation in the characteristic cystine-knot structure (50). However, in the classical vertebrate glycoprotein hormones, the beta subunits have 12 cysteine residues (50), with the two ‘extra’ cysteines forming an additional disulfide bridge that constitutes the ‘buckle’ of the ‘seat belt’ that wraps the beta subunit around the alpha subunit in the structure of the glycoprotein heterodimers, thereby contributing to their stability (50). The lack of the above beta subunit carboxyl tail extension that aids in dimerization has raised the question of whether the GPA2 and GPB5 subunits can form a heterodimer. These subunits have been shown to heterodimerize in mammals (2, 51, 52), lampreys (53) and insects (15, 16), but further investigations are required to determine whether MrGPA2 and MrGPB5 do indeed heterodimerize and to elucidate the mechanism of the heterodimerization.

In addition to identifying the genes encoding the MrGPA2/MrGPB5 heterodimer, this study also showed that the transcript of its receptor, *MrLGR1*, had a typical *in silico* expression pattern in the embryonic *M. rosenbergii* transcriptome (34). During the early developmental stages, the *LGR1* transcript in the embryo appears on day 1 and gradually increases to the highest level on day 17. Similarly, the *D. melanogaster LGR1* transcript is expressed early in development, with expression being detected as early as 8–16 h after oviposition, thereby leading to the suggestion that LGR1 may play a role in both developmental and reproductive processes (54).

The *in vitro* transcriptional study of *MrGPA2* and *MrGPB5* in the adult *M. rosenbergii* female showed similarity in the expression patterns in the eyestalk and TG, with relative transcript levels being high in both tissues. While *GPB5* has been found in the ovary of *Nephrops norvegicus* (55)*, Carcinus maenas* (56) and *P. clarkii* (11), its relative transcript level in the ovary of *M. rosenbergii* was negligible, as was that of *MrGPA2*. In the transcriptome of *Cherax quadricarinatus*, *GPA2* and *GPB5* expression was detected in both neural tissues tested but *GPA2* alone was expressed in the ovary (12). The eyestalk, TG and ovary – being major sites for the production and secretion of many hormones and receptors involved in various endocrine pathways – are the primary tissues for studying crustacean reproduction [reviewed in (57)]. The high relative transcript levels of *MrGPA2* and *MrGPB5* in the eyestalk and TG in female *M. rosenbergii* suggest that these tissues are CNS source of the GPA2/GPB5 heterodimer, which may be equivalent to the vertebrate pituitary with respect to the synthesis and secretion of gonadotropins. It is known that vertebrate gonadotropins are regulated by the gonadotropin-releasing hormone (GnRH), released from the hypothalamus (58), but, to date, the identity and function of a GnRH-like hormone in crustaceans remains subject to intensive debate (13, 59).

Nonetheless, the transcription levels of *MrLGR1* in the eyestalk and TG indicate that *MrLGR1* may play a role in feedback control in a short loop regulation, and the relatively high transcript levels of the receptor in the ovary suggest that the ovary is the target tissue. In the mammalian ovary, GPA2/GPB5 is expressed in oocytes and acts as a paracrine factor activating the cAMP cascade and the nuclear *c-fos* response in granulosa cells through the TSH receptor (52). In the adult *A. aegypti* mosquito, *LGR1* transcript expression and strong LGR1-like immunoreactivity were identified in reproductive tissues, including the testes and ovaries, which suggests a potential role for the receptor in both spermatogenesis and oogenesis (60). A more recent study (using RNAi) of the role of *LGR1* in *A. aegypti* spermatogenesis indicated that knockdown of the receptor decreased sperm yield, impaired flagellar morphology and rendered the males less fertile (19).

*MrLGR1* transcript levels were found to be sexually biased toward males in zoea 4, however it was the only case which demonstrated sexual bias. Moreover, in *M. rosenbergii*, initiation of the process of anatomical differentiation is approximately at PL10 (61), thus zoea 4 seems much earlier at a non-sexual differentiated development stage. In the present study, we aimed to elucidate GPA2/GPB5-LGR1 functional role in *M. rosenbergii* female reproduction, thus the phenomena described here are relating mainly to the adult female in which the ovary is equally developed at the previtellogenic state. Further studies should test the effects of these genes regarding male prawn reproduction processes.

Among invertebrates, activation of the LGR1 receptor by GPA2/GPB5 binding has been demonstrated in some insects. For *D. melanogaster*, Sudo et al. (15) demonstrated the role of the GPA2/GPB5 heterodimer in stimulating cAMP production mediated by the fly receptor, DLGR1. In *A. aegypti*, *GPA2/GPB5* was co-expressed in the CNS and activated *LGR1*, which exhibited ligand-dependent G protein-coupling activity (16). In decapod crustaceans, several *in silico* studies detected putative *GPA2/GPB5* GPCRs (55, 62). In the loss-of-function experiments in the present study, the transcriptional correlation between *MrGPA2/MrGPB5* silencing and *MrLGR1 *transcript levels in the *M. rosenbergii* eyestalk and TG suggests a possible ligand-receptor interaction in this decapod crustacean. To further understand this interaction, the use of additional method such as recombinant MrGPA2/GPB5 protein to activate the MrLGR1 receptor in a cell culture system could be employed. For example, Hausken et al. (53) demonstrated that lamprey GpA2 and GpB5 form a heterodimer and that a recombinant stimulates a cAMP response in COS7 cells transfected with lamprey glycoprotein hormone receptors I (lGpH-R I) and II (lGpH-R II).

**Figure 9:**
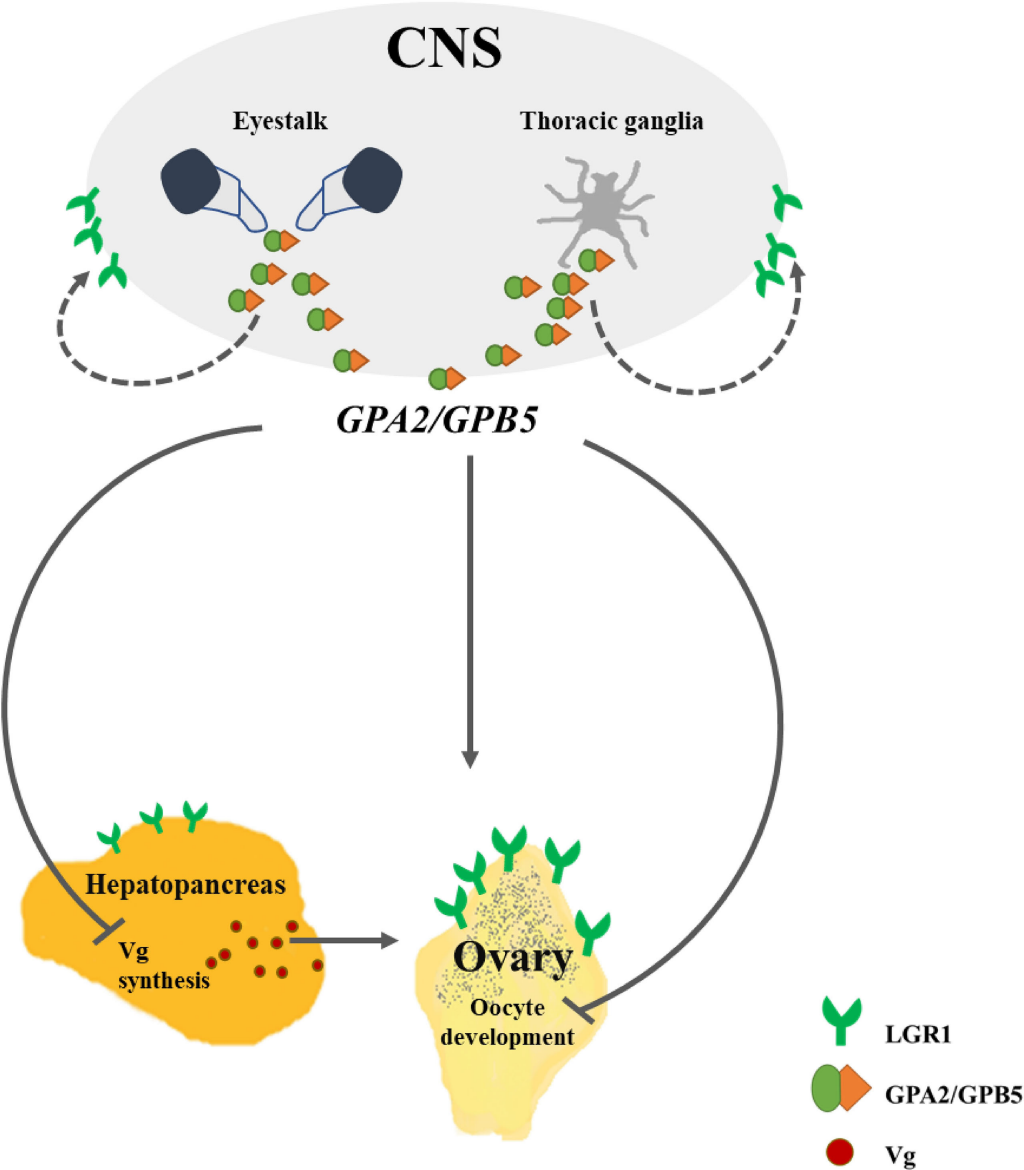
Putative model of the GPA2/GPB5 and LGR1 pathways in *M. rosenbergii* females. LGR1, leucine-rich repeat-containing G protein-coupled receptor 1; GPA2, glycoprotein alpha 2; GPB5, glycoprotein beta 5; Vg, vitellogenin; CNS, central nervous system. Solid arrows indicate known interactions; dashed arrows, possible interactions; lines with arrowheads, stimulatory effect; lines with blunted ends, inhibitory effect.

Several *in vitro* and *in vivo* studies, demonstrating the stimulating effects of the TG on ovarian growth, indicate the presence of a putative GSF in decapod crustaceans (59), and GPA2/GPB5 has been proposed as a potential GSF candidate in crustaceans (13). Contrary to the above, our findings indicate an inhibitory, rather than a stimulatory, role for GPA2/GPB5. Based upon our results, we suggest a model of the GPA2/GPB5-LGR1 system in *M. rosenbergii* reproduction (**Figure 9**). The eyestalk and TG (CNS components) serve as the site where the hormone is produced and secreted, with its inhibitory effect being exerted on a distant target tissue—the ovary. The high relative transcript levels of the receptor in the eyestalk and TG suggest autocrine regulation, through a short-loop feedback control—a premise supported by the negative correlation between *MrLGR1* transcript levels and *MrGPA2/MrGPB5* knockdown in the loss-of-function experiment.

Loss of function results suggest an inhibitory effect on vitellogenesis. The effect can directly control Vg synthesis in the hepatopancreas through inhibition of *Vg* expression; this can be supported by the significant elevation of hepatopancreatic *MrVg* transcript levels following *MrGPA2/MrGPB5* knockdown. However, *MrLGR1* expression levels in the hepatopancreas were significantly low compared to the ovary. On the other hand, *MrLGR1* knockdown in the hepatopancreas was significant. This may suggest varied ligand-receptor affinity in different tissues (e.g., the Red Pigment-Concentrating Hormone Receptor (RPCH) of *Daphnia pulex* 63). An additional regulatory effect is suggested through indirect control by inhibiting *VgR* expression in the ovary. Although the difference in oocyte diameter between the *MrGPA2/MrGPB5-*silenced and the control groups was not significant, *MrLGR1* knockdown resulted in significant elevation of ovarian *MrVgR* transcript levels and significantly larger oocytes in the silenced group vs. the control. To confirm the role of GPA2/GPB5 in vitellogenesis – and generally, in decapod crustacean reproduction – future study is needed to verify whether transcript abundance does indeed correlate with protein abundance at the different ovarian developmental stages. Moreover, additional players, such as potential second messengers (e.g., steroids, 59; intracellular second messengers such as cGMP, cAMP, and intracellular calcium, 64), are still missing to complete the full picture.

In summary, the regulation of reproduction in female crustaceans relies on a complex network that uses multiple hormonal factors, often synergistically, to control vitellogenesis and related reproductive processes (65). In the current study, using transcriptomic libraries, we identified the genes encoding GPA2/GPB5 and LGR1 and revealed their transcript expression profiles in *M. rosenbergii*. To the best of our knowledge, this is the first report of an inhibitory effect of GPA2/GPB5 in ovarian development, thereby providing evidence for the involvement of the GPA2/GPB5-LGR1 system in the control of vitellogenesis in a decapod crustacean.

## Data Availability Statement

The datasets presented in this study can be found in online repositories. The names of the repository/repositories and accession number(s) can be found in the article/**Supplementary Material**.

## Ethics Statement

Ethical review and approval was not required for the animal study because the study involves experiments in crustaceans which do not require special permits, nor ethical issues.

## Author Contributions

This study was conceived and designed by MW, TL, RM, EA, AS, and JA. MW and RM performed the *in silico* analyses. MW and TL dissected the animals and performed the *in vitro* analysis. MW and TL performed the RNAi loss of function experiments. The manuscript was written by MW and reviewed and approved by all co-authors. All authors analyzed and interpreted the data.

## Funding

This research was supported by the Israel Science Foundation (ISF) within the ISF-UGC (Grant No. 2728/16) and ISF-NSFC (Grant No. 2368/18) joint research program frameworks, and the internal research funding program of Faculty of Marine Sciences, Ruppin Academic Center, Israel.

## Conflict of Interest

The authors declare that the research was conducted in the absence of any commercial or financial relationships that could be construed as a potential conflict of interest.

## Publisher’s Note

All claims expressed in this article are solely those of the authors and do not necessarily represent those of their affiliated organizations, or those of the publisher, the editors and the reviewers. Any product that may be evaluated in this article, or claim that may be made by its manufacturer, is not guaranteed or endorsed by the publisher.
