# Supplementary material for: Genes Encoding the Glycoprotein Hormone GPA2/GPB5 and the Receptor LGR1 in a Female Prawn
Source: Front Endocrinol (Lausanne). 2022 Mar 24;13:823818. doi: 10.3389/fendo.2022.823818 (PMC8990981; doi:10.3389/fendo.2022.823818)
Supplement: Supplementary file 1 [file DataSheet_1.docx]

# Supplemental material

>*Macrobrachium rosenbergii* glycoprotein alpha-2 (accession number OL625114)
CAGCAGCACAAGTTTTTAACGTATGGTTAATACTCCAGTCTTGAAAAGAACTAAGTGTTAAAATGAAGCTTAGAATAGCCTTTGCAGTCTGGAACAAGCGGCTAGAGTAAGTAAGTATAATAACAACAGTAATAAAGATATAGTTTTATTGGGTCTGGTGAATATTAGTATCATTAAGTATAATAGTTATTTAGATCAATAAAATGTGGTCACGATTTTTTCATTGTAACATAATATCTCTATGACTTGTAACAGCTTTTTATTAATCCTCACAAATACATATTTTGATATGCTTAGAACTAATGCATTCCTTCACAACATTTGGTATGTCAATAACATGTCAATAACGGTCACAACTTTATTAGTGAGAATTTTTGTATGGGATGGGAGCTTGTGTTTGTGTAACTGATAAGGTAATGAAGTGTTTATGTATTCATACAGGCAACTTCATTCAGGTAAAGAGTTATTGCAGCATCAATATCCCAGAGATTCCAGTTTAAAAGCCGTTTTTATTTTCTCATATCTTTTTATGTTTCTTTTGCTTAATGTTTGTGTATAAACCGTTACATATAACTGCTTGTAAGATATGTAATGATATTATAAAATGTTTACGTTCAAAATTTTCATAGACTGATAACGACATGATCTTGGACGAAATATTTCTTAATGTAAATGTACGCTGAAATCCGCTAACGATCATTTACATTAAAAGTTTTTGCCGCCATAAAGAGTGCGATCGAACTGAGGCAAATGTCACATGACATTCAAGTTGAAGTCAGAGGAGAAAATAATTCTATGTTTTCAAAAGAAAGTCCATAATATGAGTAATACTAAAAATGAAAACGATAGCAAGAATATAGTTCCTTATAATTATTTTCAGAACACGACTTCGTAGTACTATTCAAGTTTTCAGTAATCCTTTAAAATCTTACGAGTATAGTATCGTCTTCGCTCTCCGTTCGGTCTAAGCCTTTTTCTAGCCCAGGCCCGAAAAGGGACGAGAAAAGGCAAACAATAAGATAGGAAAAGGGAGATGTTTGGAGCATTAGACTTGCCTTTTAAAAAAGGAAGTCTATAAGAGTACGGAACAAAAATGAGATAAAGAATAACAGATAACTTCATCCAAGTTGTATATATTTGTGCGCTACCAAGCGGGATTTTTTTAAAGAGTATTTTCTCTCAAGATTATTTGCAAAGATATTATACTACAGCAGATCACGTTTGTAATGTATAACTTTGTAATCAATTTTTGTCTCGCAAATTGGTGTCAGCATAATTGTTTGATTTTATAAGTAGAACCATATCCAATTAAATGTTCTACATTTGCTTAAAAACCTTGCATAGGATATTTTGATATATTATTTTTAAGTAGTTAGTGTTTGGGGAGATGATGTGTATTAATTGCAATTAGCGAAATTATTAATATAACCTTCTCAATTTGCCTAGACTATTTACTTCGCGACATCCAGGTGTTGTGAAGTGACGCATTTTCATCAGATTTATTGCTCAACGAAAAATTAAATCTGTACTTTGTGAGCCAGGATCCGGTATCTCCATCTCTTTTTTTTTATCACAATCATTATTTTCACTCTTCAAACCCGTCCGTCCCCTTGGTTTTCGATTTCCTTTCGTCTCTTCTCTTCTTAATACTTTTTGCAGTGGAAACAATCGCAGCTCCTGGCGGACTTGAAGATCAGCTCCCGTGGTCCGTCTATGCACATCACTTTCACTTTCACGTCTTCAGTTTCCATGATGTTGCAGCACTGGCCAATGGACGTGATGACCTGGTGAGGGTTGTAGAGAAGTGTTTGCCACGCTGAAGGAACCGACCATGATTCGCAGTATCCTCGGCAGGCGTTCGTCGTGATGTCGAATTCGACGCACTCCGGGATGCTTATCTTCCTCGTGTGCCCTACTTTGTGGCAGCCTGGCGTCTGCCACGTGTGTTGAATGCCAGCTGTGGAGGCGACGAGGCAGGCCACCAGCGTTAGCCAAGAGCTCACCATCACAGCGCTCTTATCTCCGCAGAATCAGCAAAGATACGTAGCTCAAAGTTCTCCCACTAAGTAACTGCGGTCTTGAACTTAGTTTTCTGTCTGTTTAGAAGGCAAGCTTATGAGGAAACGAATACGTCGAAACTTGAAAAAATTAAACTTGAATTGGAATGAAGTCTTTCCCAATGAATCACTGAGCAAGTCGAACGAAAGAATGACCTTTTCTCACTGGTTTTTAAACTGAACGTGCTTGGTTGGCAAGTTAGTAAACCCCTGCTCTCTCAAAGTTTATGGAATGGCGAGTGCCTTGCCCGCGTCCGCTTACCTACTTTGG

>*Macrobrachium rosenbergii* glycoprotein beta-5 (accession number OL625115)

AGAGGTGGGGGGGACGATTCCGTGTCCCCAGGGGGGCGCCTCGAGTCCCCCCGATCTGGGGGCAAAGGGAAAATGTTGACCTTGTGCGCAGGCTTGTTATTCTTCTTGGTGCTCCTGATGCCCGCAGTGGCCATCAACCCTCTCTCCACCCTCGAATGTCATCGACGTCAATATACTTACAAGGTACACAAGACTGACGACAACGGTCGAATATGTTGGGACTACATCAATGTCATGAGCTGCTGGGGACGGTGCGACTCAAACGAGATAGCAGACTGGAAGTTCCCTTACAAGAGGTCTCACCATCCAGTTTGCATGCACGAGTCGACGCAACTAACGGAGGTAACGTTACGTAACTGCGACGATGACGTCAGTCCGGGGACGGAGATTTACGCCTTTCACGAAGCTACCAGGTGTGCCTGCTTGGTCTGCAAGTCGTCTGAGGCTTCCTGCGAAGGCATACGATACCGAGGAGCCCGCCGTGCCCCTCGCGCCAAAATGCCCAGAGGTTAAAATGCCCCGCGTTCTAAAAAGTGTATATTTATTCCCAGATTGTTGGATCATTTCTTTATATCAGGAATTTTAGACTTTCCGTACATCGATTAAATAGTTTGTGCTGACACACGAATTTCAGCAACAGATACTGCAAAGAAAATCAGTCTCATTCCGGTTTTCAGCCGTAAAAGGGGGGAAATTCCGATTTAAAGTTAGGATCCAACACTCCAACTTCGAAGGAAGATTTAAGAATATCTTCTTTCAAGCTCAGGGGATTCAGAAAGAAGGTTAGAAGGCCTTTCCTTCTAGATTTCAGAACTTAAAAGAAAGGTTTAGGGATGTCTTCCTTCAAGCTCTGGGAACTTCAAAGGAACGTCTACGAATATCTTCCTGCAAGTTCAAAGGGCTGTGACAGAAGATTTAAGAATGTCTTCCTTCAAGTTGCGAGGACTTCGAAGGAAGGGTACGAATAATATCTTCCTTCCACTTCTGACTGGGAAGGAATATTTAGGAGTATCCTCCTTCAAGTTCTGGGGAACAACGGCAGCGTTACATTTCCGATCCCAAAAGTAAAAATTTGCCCTTCTATTTTAAGAAAAAATTATCTTTATTATTCGAAATCAGAGAGGTACAGTGAATATATAAGCAAAATATTAAAGTTCGACTTCTGGTGTACGAAAAACTAGAATACGCACAATACACGCCAATACCCTAAAAAAAAATTTACAGAACACCAGAGATCTTATACGCTTACCACAGGGCCAGTCGAGTAGTCACAGGTATCAAAGGCAGCAATGATGATCATCATATTTCAGAGGTTATAGTTAATACTGAAGAAAGCATATGAGAGAAATTCCTGATTATGTTTTTTTTTTAATTATTGAATAAATAGTTCAAAAGGCAATAACAGCAGATACTATCTCCGACTGTTTTTGGATTTCATTGTGAATCACTCAGATCTACTGCTAATTTCGAATACCTCATTTTCGTATACTTCATCCACCTCATTACACAAACACAAACCTACGAAACCTAATGTTTTTCATGGAAAAAGTTGTAACACCGGAGAAATACATTTTATACTTTTTATTTTATAGATGGCATGCAGATATTACTAAAATATTTGTCTTCAGTTAAACTAAAGCTAACAATTTACTAATTCAATACATTGCATACTTCTATCATATATTTTACAAAAGTAAACAAATGCACAAATAAAGCTAAATCGTTCTATTTTTTTTGTCAAAGATACCATTGCAAGACGCGACAAATGAAGATGATATCCATTTAAGCGTACTTTTTATGGACAACGTAATATTAGTTTTAACAAATGAATGGGAATGTTCTGTGACCTGAGCTGCCTACTTTCGATTTTTTTTAATCCTCTAAGACATTTCATTCTATTAATTTCTGCACACAATATATATATATATATATATATATATA

>*Macrobrachium rosenbergii* leucine-rich repeat-containing G protein-coupled receptor 1 (accession number OL625116)

TGCTGCTGCTGCTGCTGCTGCAGCTGCTGTTGGCAATACTCTGAGGTATCATCGTCGCTGTGGTTGCTGTGGCTCATCTCCTGCACCGTACTTAGCCGTTCAGAGTCTCCAGTACACGTCTCTGGCTTGGTTGGGGTCCTTGAAGGAGAGATGTTTTTCTGCAGTGAGTCCTGGGATCCACAAATGGCTGTTCCGGGAAGAGAACCGCTGTTGCTCTGCTGAGAAGTCCTACCAGCCCGAGTCAGGTCACAAAGGGTGATTTGTGTCAACGTAGAGTTGCGGTGATTGGGACCAGCGTTGACAACGGTGTTGTGAGGAAACGTGTTATTTACGGATGAATATGCTCCTTTGTACCTCATGGCTTTCTTAGTGCAAAAGCCGTATCTTGCCAAGAGAATGAAGAGATCCCTTCTGTACTGCTTCGTGAGAATGGCGTACAGATAGGGATTGGCGCAAGAGTTAAGGGGATAGAAGAAGACCAGTAGAATTTTGGCTCTCGTCACGTTGATAAGTGGTATGCCAGCTACTGCCGTCAACCCAAAGAAAGCGATGGGCGCCCAGCAGGCAAAGTCTGTGAAGACGAGGAGAGCCATTCTCTTGGCTACGGTGAGATCGGAGTGAGACGCAGATGCGTCGTGCCTGCTGATTGAGCAGTACATGGAGGCGTAACAAGCGGTTATGACGATGAAGGCCAGACCGTTAACCAGCAGGAGGGAGATGAGGTACGTGAGGGCCAAGCCGTTGCCCGTTTCCATTGGTAGGCAGATGCTTGTTTTGCTATATCCGGAAATGCCGATGAGGGGCAGAGTGGCCATCGTAATGGAGTAGATCCATCCGATGACCATAATCTTGGCAGCCATGGAAAGCTTGAGCCTCTTGTTGAGGTGGATGGCGTAGGTGATGGCGTACCACCGTTCCGACGTGATGATTGTCAGCGTGAAAATGGACAGCTCGGAAGCGAACACTGTCAGGAAGCCCGCCACTTTGCAACCCGGACCATTCTGCCAGTCGATGGCATAGTTGAAATAGACACCGATGGTGTGAAGGTCCATTGAAGCAATAATCAGGAGATAGATTCCCATGCTAAGGTCGGCGAGGGCAAGGTTCACCATCAAGAACTTGCTCACTGTCATCCTGAATTTCGAGGAGACCAAGACAAGCATGACGGCCAAATTGCCAACAACAGCTGTGACAACCACAAACCAGACAGCAACGCGAAGAGCGATGTTTCCCATGATGTCCTCGCAGGGGTTGAAGGCGTCAGGAGCGGGGACGCACATAACATCATGGTAGTTTCTCGAGAGGTTTCCACAGAGCACGATGATCGTTGTATTATTCATAGGGGCCCTCGTTACATGCCATTTGTCGTCCTCATTCTTTAGATTTGTGTTTGGAATGTCCTCGGAAAATATCTCTTTTTCGACGAACCCTCCTTCATCAATCAATCCTTCTTCAGGGTAACCAAAATGCTCCGGGGAATCCCCGTGACCTGGCCAAATCTCGGGATCTCCCCAAACGCCGACAAGAGTGTCATTTTGCTTGAAAGTATCCGATGGATACCTATCAGCTGAGTGATTGAAAGGTCCAAAGCCAAGGAAATAATTCTGGAATCCTCCTGGACCTCCGGCCCCTGCCTCGGCCATGTTAGTATCTAAGATTCCTCCCGGGTTTAAGGGAATTTCATCTGTCCCAAAGCCTTTATCTAGGATAGGGTCGAATGCCTCTTTTGATAAGGGATCCCCAACTTCGGCACTTTTTGTTTCTGACTTCTTATTCAGAATAGGAAGGATTTCCATCGACTCCTTCAGTCCAAAACCATTTCCTACACTGCTCTGTGACCCCTGAAATAATTCCCTCCTGCTCAGCCTGGCATGCTTAACTGCGTGAGATCCTTTTGATGGCTCAAACCTCTGTTTCATTTGATGTCTGAGAGGAATTAAATAGCTACTTTTGATTTCTTCATGTTCCACACTTTTAGATTTGTCAGATTCGGGGGTGGTTATATTTACATCTGGCCTTGAACGTCTATGTCGTGAAACATAGTGTTTTGCTTTTGAGGTAGGCATTACTTCATTCACATGTACGGATTTACTGCTTCTTTTAACTCTGTTGCTTGTAGGCAACATTTTTGCTAAATCAAAGTTTTCAACAGAGTTCATGAAGGAGGACTTGGTGTCTGGAAATCGCCCAAATGAATCAACAGGATAGGAAGCTCTTGGGAGCTGATTCCAGTGCGTTGCCTCTTTACGAGCTGACTGGGTCCTTGAAGTAGCGTACTTTTTCCTTCCAATCAATGACTCTGGCTGAACAGAACCCCAACTTCCATCTTCAGAAGAGGAAAAGTCTTGATAATTCATGTGTTTCTCAGCCACTGCCCCAGTATTCTGATAGGATGACGACAGGGAAGTCAGAACGCCTTCTACAGTGGGCGCAGTAACGGGCTGGATGGGTGCGTACCGCCGCTGTCTGATACGTGTGGTTTGAGTCATGGTCGGTGATGTCGGGGACGTCAGCTCAGAGCAGTGTTCCTGTTCCATTTCTTTTAGTTTCTGGTGGATGACGTACTGCTCCTTTTTATGCTGCTCGGGAAACTGGAAGGCGCAGCAGTGGTACGGGTAAGTCAAGTGCGCCTCCTTAATCGTTCTGAACGAGTAGACAGAAGGGAAGACCTTGAGAGTTGGAGTGTTCTCCAGTCGTAGGACTTCGAGTTCCTTCAAGCCGACCGTTGGCAGGGCTGTTATGGACGTCTCCGACAGGTCGAGTGTCCTTAAGCTTCCAATGCCTTCAAAAGCATCCGGATGAAGATAAGTCAGTTCCTGGTTTCCCTTGAGGCTTAGTTTTCCAATATACGAGCCCGAAAAGGCCTCTCTTTCCACTTGCTGGATGTGGTTATACCCAAGCGACAACTCCTCTACTCGAACCTGTAGGGACCGGCTGTGGATCTTCTGAATCCTGTTACTGTCCAGCTCAATCATGTGAAGTATCTCATTACTGTTGAACCGTAGATTAGGAACTGCTTCTAGGCCAGAACTCACAATCCTCAAACTTCTCAGTTTGTCCTCAGTAAAGGTGAGATTTCTGATGAACTTCAATTTCGGAGCCGAGTAAATGTATATTGAACGTAGTTTAAGAAGCCCACTGAAGCATCCGGCTTCGATTTGGTGAAAATCTTGAACGTTGTGAAGAACTACATCCCGGAGCGTCGCTCGGTATCTGGTGAAGGAGTCATTCCTGAGGGTCTCCAAGCCAGCATTCATCACGGTTATTCTGAACACCTCCTCCGAGAGATTAGCAGGAATAGACTTCATGTTTGGCCCAGAGCAACTGCACTCTACCTCTGGGCGGGGGGGATCCGTCCCCCAGCAGCCGCAAGTGGAGTCACTGTTCTGTCCAGGAACTGCAAGGCTTTGCATTGGGAAGTCATCCATGTTAATCATCCGATTATTGCCTCCATACTGGTGAGAGTGGTCTGATTCTCCCCAATAGTCGTCTGGGTCATAATAATCGTCGTTCCTCTCACTTGCGTCATAACCCTCCAGTGGATACCATTCCACAGGGTTTTTTGTATATTCGCCTGCATAAATGCGACTGCTGTTTTTGACCAGAGAGATATTCGTTGCTAGGTTACTTTCTGGGTGGCTGTAGTTGTTTGCTCCTGCCATCTGATTATCCATATTCTTATTCCTAGCTTTGCTTAATTTATCATCTAGGTTGCGTCTTCCTCGACCTCCCACCTTTTCTAAATAGAATTTTTTTTCACTACTTATATGAGAGATCGAGGAGTTCCTGATGTCCCCGTTCTCTTTCCTTGAGGATTTATGTGGATGGTTGTGTTTTCTGTATCTCCATCTGATGTTCGACAAATAATGAGGGGTTGTTAACTTTTGCCTCCCTGCATCTGAAGATGGAATATCCCGCGGTGAAGCTAATCGATCATGGTGATTAAAATGGCTTCCCTTCAAAACATGGTTCTCGGTGCCATCCCTACTTTCCTGAGGTGAATCCTTAATAACGTCGTCTGTCGAAGAGTTCTCCGACAGCATGCGAGGATTAATTAAAATTTCTCCAAGTTTCCCCCCATCAGTTTTCCCTAATGGGAAAACCCTATCTCTCGGCAATGCTTCTTGAAATTGGAAATGAGGAGAGGCCTTCTTGTTTGCCGAAATCGCCCTTCCCTTTCCTCGGGGAAAAGACCCCTCGCGTTTCCCGTTTTTCTTTTGATTAAAGCCCTGTTGCCCTTGACCACCAGCCCTTCCTGAACTTCCAGCCCGTGCTACTTCCGCCTTGGTTAGTTGGCGGCTGTATGTTCTTCTCGCCTCCGTTTCCATAATGGAGAACCCGCGTTCGAGTGCCTTATCCCCTTTGGCGGCGTTACTCACTCCTCCCCTTAATGATATTTTCTCGAATTCGAGTTCCTTGTCATCCCCTCGAAATTCTCCGGCCTTGCGCTCGTTGCCGAGCTTCCTCTGAGAAGCCTCTTTATTGTTCGTGCTCTTTGGGAAACTTCTTTCTTGTTCTCCTTCGCCCCCTCCTCCGTCGACCCCTCCTTTCTGTTTGTCAACATTTTGGAAATGCGTCACGTGGCTGCTGCTTTGCTCGCCATCGTATCCATGGCTAACCTGCAATGCAAGGCTGATTTTCTCTCTGGTCGTCAGATAATCATTTATGGGCTGGGGATTATTGTGCTCAAGAACGGCTACCCGGGGGTCAATCAGGACAGCATTTCGACCTTTTTGTGGGGTTCCCGGGATTTCTTGAGCCATCAGCTCATCTGCGAATAGGTTCCCATCGTTAGCTGACCTCTGCAACTCAACAGCAGCTGCGATACTGTAGAAGTTGAGCATCACGAGTAACTTGAGTAACATTATCTCCATCCCATGGGATGCTGGGCAACCCTTCGTGGCTTTCATCATCAGTGCTCACATTTCTGGTATTTTGCTTATTCATGGATGAGTAGCATCGCCAACATCTTAGGCAGTTTCTCCCTGAACAAAGGTTTCGCAGAAGAAAAATAAAATAGCAAGGTGGAATAGCACCGATGCTCCTAATTAACGTAATGTTGTCCAAACATTCCCGCTTAATGGTGAGCTTTTTAACCTGGTCATGTTAGTTGGTCTTACACTTTGTTTCTTTTGTTCAAAATCATCTTGCCTCTCTTCAGAGGAAATGACATAAGTCACGAAAGCTCCTCAAAGGAAAGGTGTACAAAACAAAGGCCGTTAACCGAGCGCCTATCGGTTGCGAATTTTCTTGCGAGGGGAAAGAGGGGAGTTTTTCGGGAATCACACTTTGCACTATGTCAATATTGGGACGCGCGGCCACGGGCGACGACGCCCGCCTTCCCTCACACTAGTAGGGCTGACCCTTCACTGCTTAGCTGTCAGCTATAAT
